# Encapsulation of Exosomal Volatile Organic Compounds for Modulating the NF-κB Pathway in Monocytes

**DOI:** 10.1021/acsbiomedchemau.5c00241

**Published:** 2026-03-16

**Authors:** Dina Hashoul, Rand Shibel, Haya Dahamshy-Silawi, Elias Mansour, Nisrine Lahoud Jeries, Walaa Saliba, Yoav Broza, Assaf C. Bester, Hossam Haick

**Affiliations:** † Department of Chemical Engineering and Russell Berrie Nanotechnology Institute, Technion—Israel Institute of Technology, Haifa 320003, Israel; ‡ Faculty of Biology, Technion—Israel Institute of Technology, Haifa 320003, Israel

**Keywords:** VOCs, inflammation, immunomodulation, exosomes, NF-κB, communication, liposomes

## Abstract

Cytokines communicate and coordinate immunity, allowing the immune cells to understand one another. Aside from cytokines, a wide variety of molecules modify immune cell behavior and immune responses. We present a study of volatile organic compounds (VOCs) and their capacity to influence the NF-κB signaling pathway in monocytes. By using the U937 monocyte cell line and CRISPR/Cas9 gene editing, we created a model of NFKB1–/– human monocytes. The results showed that exosomes from distinct cells and the same cell have unique VOC signatures when stimulated by LPS. To test the effect of certain VOCs on NF-κB, we encapsulated them in nanoparticles (liposomes). An example is 2-butanone-encapsulated liposomes, which reduced NF-κB and lowered the level of proinflammatory cytokines (TNF-α, IL-8 MCP-1, and IL-1β) in LPS-stimulated monocytes. These results suggest potential therapeutic targets for modulating the NF-κB signaling in monocytes.

## Graphical abstract

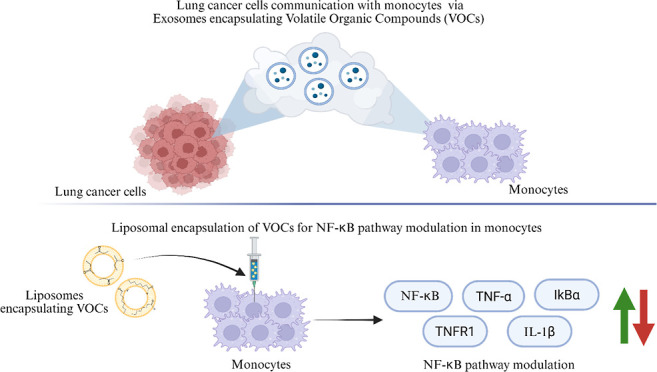

## Introduction

The Nuclear Factor kappa-light-chain-enhancer of activated B cells (NF-κB) is a pivotal transcription factor critical in immune response regulation, cell proliferation, and survival. Its importance is especially evident in the pathophysiology of lung diseases, where it orchestrates a complex network of inflammatory and immune responses.^1^ The NF-κB pathway is a central player in lung conditions, such as chronic obstructive pulmonary disease (COPD), asthma, acute respiratory distress syndrome (ARDS), cystic fibrosis, and lung cancer. It contributes to the perpetuation of inflammation, tissue damage, remodeling, and, notably in lung cancer, the promotion of tumor growth and resistance to apoptosis.^2,3^

Given its pivotal role, managing NF-κB presents viable therapeutic options for addressing these diseases. Inhibitors targeting NF-κB have demonstrated positive outcomes in both preclinical investigations and clinical trials.^3,4^ Various immunomodulatory strategies have been devised as therapeutic adjuvants for mitigating lung inflammation, encompassing small-molecule agents, monoclonal antibodies, and other pharmaceuticals.^5^ Certain treatment modalities, such as canakinumab (an interleukin-1beta inhibitor) and anakinra (an interleukin-1 antagonist), show therapeutic advantages in modulating hyperinflammation, resulting in decreased mortality rates in clinical settings. However, these modalities necessitate high doses and frequent administrations, typically resulting in systemic side effects.^6^ Additionally, to detect NF-κB activity in lung diseases, researchers and clinicians use molecular biology techniques such as Western blotting, electrophoretic mobility shift assays (EMSAs), and immunofluorescence. Although informative, these methods have limitations in sensitivity, specificity, and real-time monitoring of the NF-κB activity in living organisms. To address these limitations, sophisticated, minimally invasive methods are needed to detect and treat NF-κB-related lung diseases. Therefore, additional efforts are required to devise an effective strategy for regulating lung inflammatory pathways.^6–8^ Innovation in nanotechnology, biomarker discovery, and targeted drug delivery systems holds great promise in developing novel diagnostics and therapeutics for patients suffering from NF-κB-related lung diseases. The ideal approach would allow for the precise and real-time monitoring of NF-κB activity within specific cells or tissues in the lung coupled with targeted therapeutic interventions that can modulate this pathway with minimal systemic exposure. This advancement could lead to more effective, personalized, and less invasive patient treatment options.^9,10^

Studies have demonstrated the activation of diverse immune cells during lung inflammation. Specifically, monocytes, the progenitors of macrophages, serve as the frontline. Cells of innate immunity not only identify pathogens to generate cytokines but also release inflammatory factors to modulate the adaptive immune system. This, in turn, triggers the onset and progression of inflammation.^6,11^ Recent research suggests that volatile organic compounds (VOCs), traditionally considered mere byproducts of cellular metabolism, can serve as significant players in cell signaling and intercellular communication^12^ in monocytes. Our findings suggest that specific VOCs can be intricately linked with the activation and regulation of NF-κB in lung diseases. By detecting these VOCs, we may develop a novel, noninvasive method to monitor NF-κB activity, offering a real-time snapshot of disease progression and response to treatment. More importantly, our research points to the potential of manipulating these VOC-mediated signaling pathways as a therapeutic strategy. By disrupting or modulating the communication mediated by these VOCs, it may be possible to attenuate the aberrant NF-κB activity characteristic of many lung diseases. This strategy represents a significant shift from conventional treatments, moving toward a more targeted approach that could mitigate the pathway’s activation without the need for systemic drug administration. Such a method could dramatically reduce potential side effects and provide a more patient-friendly therapeutic option. Our exploration into the role of VOCs in NF-κB signaling challenges existing paradigms in pulmonary disease management and paves the way for innovative treatments that align with the principles of precision medicine. Thus, this discovery of the role of VOCs as signaling molecules in the NF-κB pathway could potentially open new avenues for both detection and therapeutic intervention, which could revolutionize the field of pulmonary medicine.

## Results

### Exploring the NF-κB Pathway in Unidirectional VOC Communication

We aimed to examine how the VOCs emission from the A549 cancer cell line may affect the NF-κB pathway in human monocytes of the U937 cell line using a specially constructed microfluidic chamber. The 3D-printed glass chamber features two separate wells connected by a one-way valve created by Tesla (Figure 1a) in a consistent structure. This makes it possible to investigate the unidirectional and noncontact communication between two different cell types.^12^ We devised a communication system where cancer cells were placed in one well and monocytes in the other (A549 → U937), as shown in Figure 1a. VOCs released by cancer cells were transmitted to the headspace of the monocytes through the Tesla check valve and were accumulated in the adjoining well. The gas environment above the U937 cells was continually monitored by drawing samples from the headspace using Tenax TA sorbent tubes (Figure 1a). To investigate the intended impact of VOCs on the NF-κB pathway, cells were collected every day, and the relative levels of the NF-κB signaling proteins were analyzed. The relative amounts of TNFR1, *p*-FADD (S194), *p*-NFκB (S536), *p*-IKKα/β (Ser177/Ser181), *p*-IκBα (S32), and c-Myc were evaluated in the A549 → U937 arrangement. Phosphorylation levels of *p*-NFκB (S536) and *p*-IκBα (S32) both showed a similar trend in the four-day culturing period (Figure 1b): there was a quick upsurge after 1 day, which was followed by a progressive reduction across the second and third day to reach a similar value to the control group (Media → U937). Phosphorylation of *p*-NF-κB (Ser536) increased approximately 2-fold after 1 day of VOC exposure (*p* = 0.014) and subsequently declined, reaching levels approximately 5-fold lower than controls by day three (*p* < 0.001). Similarly, phosphorylation of *p*-IκBα (Ser32) increased after 1 day (*p* < 0.001) and decreased markedly by day three (*p* < 0.001).

**Figure 1:**
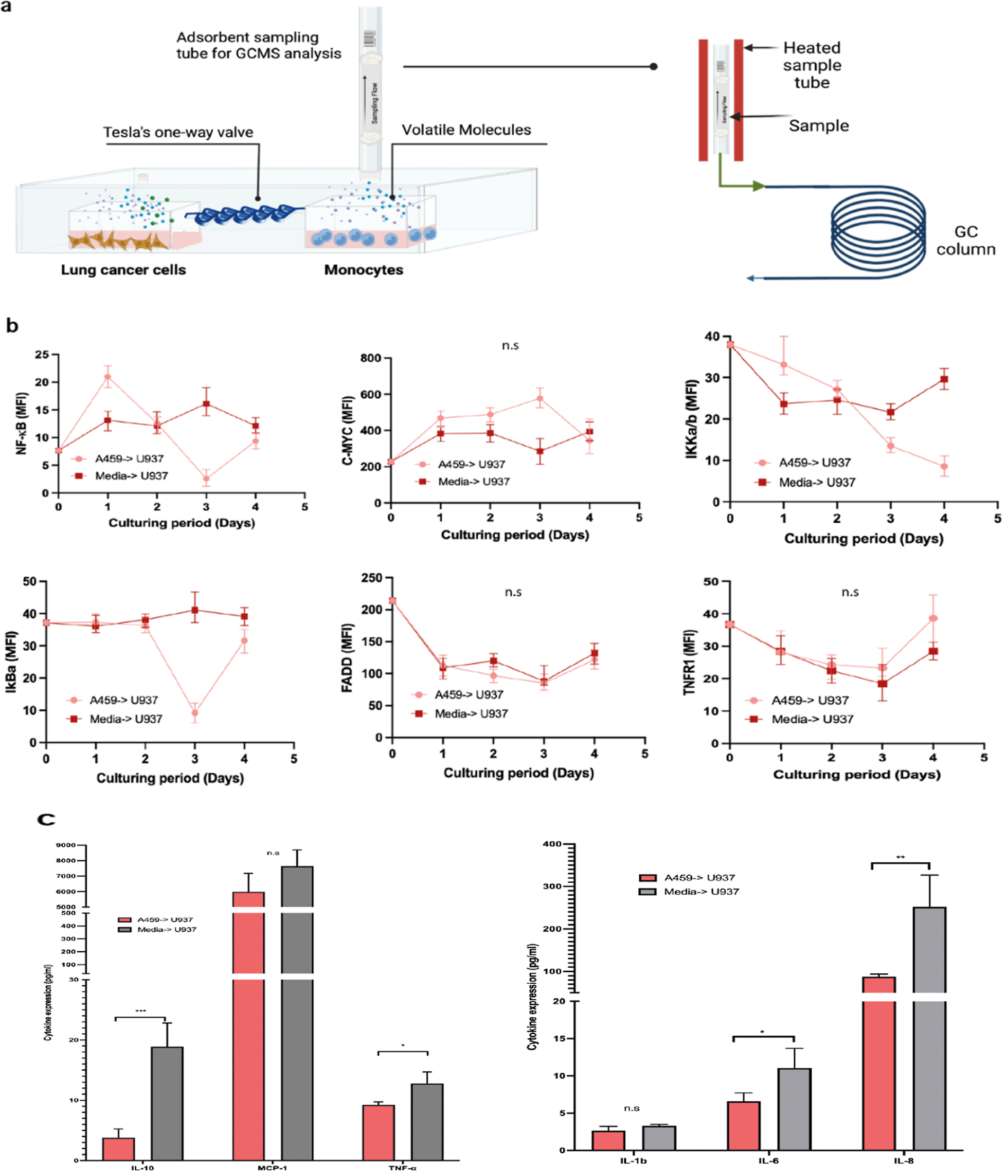
Unidirectional volatile communication effect on NF-κB activation. (a) Schematic illustration of the unidirectional communication setup. (b) Relative proteins and phosphorylation levels of TNFR1, *p*-FADD (S194), *p*-NFκB (S536), and *p*-IκBα (S32), and relative protein levels of c-Myc during the unidirectional volatile communication in U937 cells. (c) Cytokines levels of IL-10, MCP-1, TNF-α, IL-1b, IL-6, and IL-8 in U937 after 4 days of incubation with A549. Data are means ± SEM (*n* = 4) and were analyzed using Student’s *t*-test (*p* < 0.05).

(Figure 1b). Additionally, the levels of *p*-IKKα/β (Ser177/Ser181) varied over the course of the culturing period, increasing on the first and second day (*p* = 0.02) (Figure 1b), before reducing across the remaining days to eventually demonstrate values lower than the control group (*p* < 0.001). Meanwhile, there were minor dissimilarities between the communication setups for the remaining proteins measured (TNFR1, *p*-FADD, and c-Myc) (Figure 1b). The changes and differences in the regulation of the NF-κB-related proteins indicate that VOCs can both switch on and repress the NF-κB pathway based on the duration of the culturing period and the concentration of the collected VOCs in the monocytes’ headspace.

To analyze the effects of VOC communication between lung cancer cells and monocytes, we studied the concentrations of various cytokines associated with the NFκB pathway (IL-10, MCP-1, TNF-α, IL-1β, IL-6, and IL-8) at the end of the culturing period (Figure 1c). We observed decreased IL-6, IL-8, and IL-1β which are produced due to activation of the NF-κB pathway and decreased TNF-α which activates this pathway.^2^ While activation of NFκB has both stimulating and inhibitory effects on IL-10,^13^ we observed significantly lower levels of IL-10 in A549 → U937.

### Defining the VOC Signature of *NFKB1* Knockout U937 Cells

To understand the unique VOCs associated with the NF-κB pathway, we used CRISPR/Cas9 gene editing to develop an NFKB1-deficient U937 human monocyte cell (Figure 2a). NFKB1 removal influences the NF-κB pathway by altering its activation and the related downstream signals.^14^ Its absence has been linked to greater activation of the NF-κB pathway in certain circumstances, for example, autoimmune illnesses, T-cell lymphoma, and melanoma.^15^ As it is the most plentiful transcription factor in monocytes and macrophages, which are central in inducing persistent inflammation, NFKB1 not only directs TLR-initiated proinflammatory gene expression in activated macrophages but is also involved in determining macrophage polarization and innate immune memory reactions.^16^ Taken together, this demonstrates that deleting NFKB1 can lead to higher NF-κB pathway activation and modified downstream outcomes in many cellular activities and illnesses.^17^

**Figure 2:**
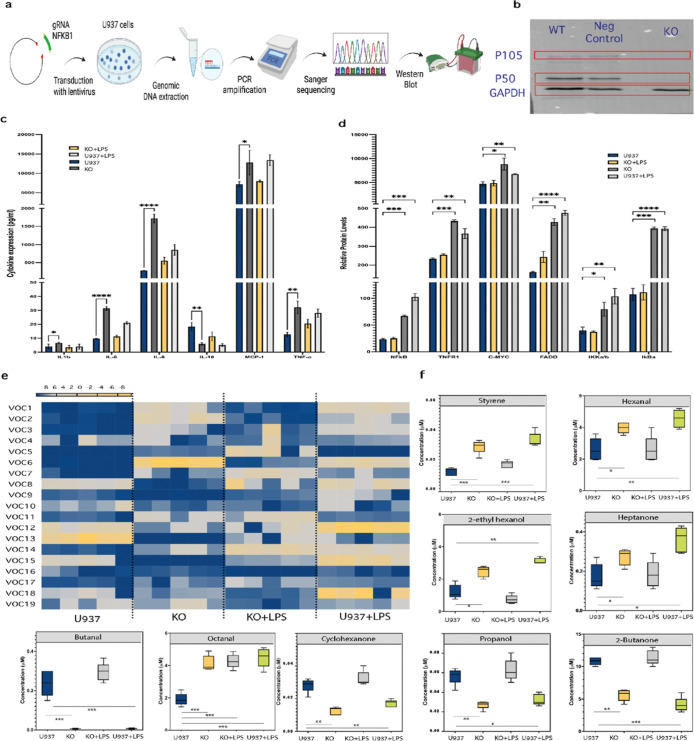
Volatile signature of NFKB1 KO in U937 cells. (a) Schematic illustration of the generation of NFKB1 knockout in U937 cells using CRISPER/Cas9 gene editing. (b) Levels of p105/p50 (NFKB1 protein products) in WT (naïve U937) and NFKB1-deficient U937 cells (KO) analyzed by Western blot. (c) Cytokine levels of IL-10, MCP-1, TNF-α, IL-1b, IL-6, and IL-8 in U937 cells and KO cells. (d) Relative proteins and phosphorylation levels of TNFR1, *p*-FADD (S194), *p*-FADD (S194), *p*-NFκB (S536), and *p*-IκBα (S32), and relative protein levels of c-Myc in U937 and KO cells. (e) Heatmap of significant (*p* < 0.05) VOCs. Each row represents the peak area of each compound in U937 and KO cells. VOC 1: benzaldehyde, VOC 2: methyl-vinyl-ketone, VOC 3: butanol, VOC 4: heptanone, VOC 5: 2-ethyl-hexanol, VOC 6: butanal, VOC 7: pentanal, VOC 8: butanol, VOC 9: 4-methyl heptane, VOC 10: styrene, VOC 11: cyclohexanol, VOC 12: 9-methyl-1-decene, VOC 13: octanal, VOC 14: ethyl benzoate, VOC 15: tetradecane, VOC 16: hexanal, VOC 17: propanol, VOC 18:2-butanone, VOC 19: cyclohexanone. (f) Box-and-whisker plots show the distribution of abundance in the concentration value (μM) of nine selected VOCs in U937 and KO cells. U937 and KO cells were stimulated with LPS, and the supernatant was analyzed (*n* = 5) for GCMS and (*n* = 3) for cytokine and NFkB measurements. Data are means ± SEM (*n* = 4) and were analyzed using Student’s *t*-test (*p* < 0.05).

Utilizing targeted CRISPR/Cas9-guided double-strand breaks, we created a five-base pair deletion in exon five of the NFKB1 gene, which induced a premature stop codon. This absence of p105/p50 protein was then confirmed by Western blot analysis, Sanger sequencing, and qPCR (Figures 2b and S4). We further evaluated the influence of NFKB1 knockout on the NF-κB pathway activation, which was assessed by measuring the phosphorylation of the p65 subunit of NF-κB at S536 by the IKK complex-IKKα/β (Ser177/Ser181).^14^ The results were consistent with those obtained from NFKB1 knockout THP-1 cells, indicating that NFKB1 is not essential for IKK activation in the NF-κB pathway (Figure 2c). To analyze the function of NFKB1 in controlling inflammatory gene expression, U937 and KO cells were activated with LPS for 8 h, after which Multiplex analysis was conducted for cytokines such as IL-10, MCP-1, TNF-α, IL-1β, IL-6, and IL-8. We observed decreased IL-10 expression in NFKB1 KO cells, which mimicked the results of individuals with NFKB1 haploinsufficiency and NFKB1–/– THP-1 cells.^17^ Moreover, stimulated KO cells showed reduced IL-10 levels compared with WT controls (Figure 2d).

Two main analyses were conducted to evaluate the effects of the NFKB1 deletion in KO cells on their VOC signature. First, the VOC signature of KO cells was analyzed and compared to a cell-free medium from all respective cells to determine their metabolism under resting conditions. Second, the VOCs’ output of U937 and KO cells was compared to resting U937/KO when exposed to LPS to understand how LPS modifies the monocyte VOC metabolism.

A Wilcoxon signed-rank test showed that levels of 19 VOCs were significantly expressed between KO and U937 cells. Compounds were identified in two steps. Initially, the peak spectrum was cross-referenced against the NIST mass spectral library. The identification was then confirmed by examining the respective retention times and substances’ specific *m*/*z* ratios of most significant compounds compared to retention times acquired for reference mixtures made using the method section. All significant VOCs (*p* < 0.01), based on the average peak of a total of five chromatographs for each sample, and their statistical significance, are shown in the heatmap in Figure 2e. Additionally, the concentration of 9 VOCs was determined using a standard curve that was prepared with analysis by GC-MS. Standard curves were induced using the peak area. Calibration measurements were used to determine the limit of detection (LOD) under the experimental conditions of our instrument and the average concentration levels of 9 VOCs. Figure 2f and Table S1 summarize the average concentration levels and the calculated LOD of the 9 VOCs in micromolar and ppb concentration. Among the significant VOCs (Figure 2e) were five alcohols, four ketones, three alkanes, and four aldehydes. These compounds included octanol, 2-ethyl-hexanol, propanol, cyclohexanol, and butanol for the alcohols, 2-butanone, 2-hexanone, cyclohexanone, and heptanone for the ketones, styrene, tetradecane, and 4-methyl heptane for the alkanes, and octanal, pentanal, hexanal, and butanal for the aldehydes.

In resting cells, KO cells demonstrated increased amounts of aldehydes (hexanal, butanal, and octanal). The ability of monocytes to metabolize aldehydes helps moderate the proinflammatory effects of certain aldehydes, which have been verified to promote monocyte adhesion, cytokine production, and the intake of oxidized lipids.^18^ Three distinct enzymatic pathways may facilitate aldehyde metabolism in monocytes: the oxidative activities of aldehyde dehydrogenases (ALDHs) and cytochrome P450 (CYP450); the activity of glutathione S-transferases (GST); and the reductive activities of aldose reductases (AR).^19,20^ According to our data set, the aldehyde octanal was found to be higher in LPS-stimulated and KO cells (Figure 2e,f, Supporting Information Table S1) (*C*_KO+LPS_ = 4.1μM, *C*_U937+LPS_ = 4.35μM, *C*_U937_ = 2.13 μM, *C*_KO_ = 4.03μM).Interestingly, the enzyme responsible for octanal metabolism in monocytes, ALDH-2, was found to be significantly lower in monocytes and peripheral blood mononuclear cells (PBMCs) during chronic inflammation.^21^ Also, studies have established that oxidative stress can alter ALDH2 activity by forming reactive oxygen species (ROS).^22^ ROS can alter ALDH2 and influence its enzymatic activity and stability. Inflammatory cytokines and signaling pathways linked with NF-κB activation can influence ALDH2 indirectly by influencing its transcriptional regulation factors or other downstream signaling pathways.^21^

We observed an expansion in ketones in WT cells compared to KO and LPS-stimulated U937 as observed in 2-butanone and cyclohexanone (2-butanone: *C*_U937_ = 11.46 μM, *C*_KO+LPS_ = 12.34 μM, *C*_KO_ = 6.01 μM, *C*_U937+LPS_ = 4.85 μM, cyclohexanone: *C*_U937_ = 0.03 μM, *C*_KO+LPS_ = 0.036μM, *C*_KO_ = 0.01 μM, *C*_U937+LPS_ = 0.017 μM) (Figure 2e,f, Supporting Information Table S1). Ketones are products of fatty acid metabolism, and acetyl coenzyme A is utilized as a precursor to participate in numerous metabolic processes.^23^ Interestingly, during inflammation, in addition to NF-κB activation, monocytes go through metabolic modifications to support their immune functions, for instance, augmented fatty acid uptake,^24^ increased fatty acid oxidation entails the degradation of fatty acids to produce acetyl coenzyme A.^25^ Both 2-butanone and cyclohexanone are linked with pulmonary diseases such as chronic obstructive pulmonary and asthma, among others.^26,27^ On the other hand, the ketone heptanone was found to have greater levels in LPS-stimulated cells than in both WT cells (Figure 2e,f, Supporting Information Table S1).

Exosomes from NFKB1-deficient monocytes display a distinct VOC profile. VOCs originate from various chemical families and have varied physicochemical features that dictate their behavior in humans.^28^ For example, fat–blood partition coefficients influence the concentrations of VOCs in air, fat, and blood, impacting their absorption, dispersion, and removal from the body. Moreover, they are characterized by high volatility and lipophilicity, meaning they cannot be transported directly into the bloodstream. Therefore, it is thought that they could be held in exosomes, which are extracellular compartments that act as conduits for the passage of bioactive compounds like proteins, lipids, and nucleic acids between cells. Exosomes, measuring 30–150 nm in diameter, can move these molecules across short and long distances and influence the behavior and characteristics of the recipient cell. This is important for cellular activities, such as the control of immunity. Therefore, there is potential for a relationship between exosomes and the transport of volatile molecules, suggesting that they could play a role in long-distance intercellular communication.^29^

To assess our hypothesis, we separated the exosome fraction from the WT, KO, and A549 cells. The monocyte/cancer cell medium was collected and spun down to remove intact cells and cellular debris from the conditioned medium. Subsequently, the clarified medium contained soluble factors, MVs, and exosomes that were then centrifuged for 4–6 h (Figure 3a). The isolated exosomes were evaluated with DLS and SEM, which ascertained their regular spherical shape and size (124 + 14 nm) which is normal of exosomes (Figure 3b). Furthermore, we inspected the exosome fraction (*n* = 7) using ITEX-GC-MS to find out their VOC signature^29^. The color map in Figure 3c displays the unique volatile print of the exosome fractions from the different cells. A total of 8 VOCs were identified to be distinguishable among the mentioned cells, with four of them discerning only KO/LPS-stimulated, WT controls, and A459. Additionally, the other four were specific to A549 cells (Figure 3c). Specifically, 2-ethyl-hexanol was observed to be greater than all exosomal fractions derived from monocytes, in keeping with other research that indicated increased levels of 2-ethyl-1-hexanol in the lung cancer line, NCI-H2087 and A549 in vitro.^30,31^ In a manner similar to the volatile analysis carried out on the conditioned medium, octanal, 2-butanone, 3-hexanone, and octanol were also significantly altered (Figure 3c). We evaluated their concentrations in the exosomal fractions with pure standards and calibration curves (Figure 3d, Supporting Information Table S2). Octanal was greater in LPS-KO and LPS-stimulated U937 exosomal fractions (*C*_U937_ = 0.24 μM, *C*_KO+LPS_ = 1.65 μM, *C*_KO_ = 0.44 μM, *C*_LPS+U937_ = 2.03 μM, and *C*_A549_ = 0.61 μM) (Figure 3d, Supporting Information Table S2).

**Figure 3:**
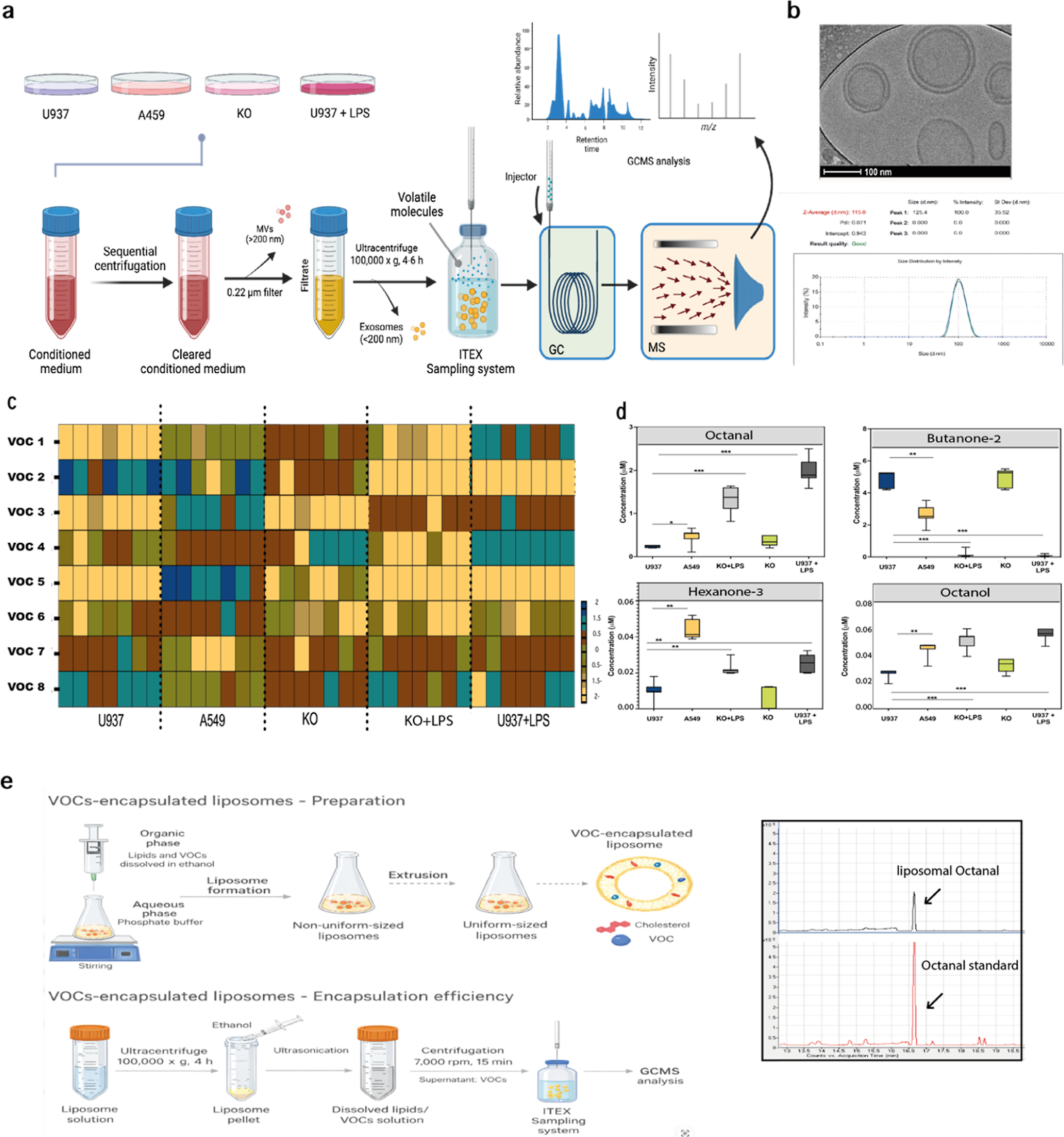
Volatile profile of exosomes isolated from U937 and NFKB1-deficient U937 cells. (a) Schematic illustration of exosome isolation from cells and subsequent GCMS analysis. (b) Morphology and size of exosomes observed using transmission electron microscopy and dynamic light scattering (DLS) number distribution measurement of purified exosomes showing a single peak at ∼120 nm. (c) Heatmap of significant (*p* < 0.05) VOCs. Each volatilome has five biological replicates; each column represents one biological replicate (*n* = 7). Each row represents the peak area of each compound in the exosomes fraction isolated from U937, KO cells, and A459 cells. VOC 1: octanal, VOC 2: 2-butanone, VOC 3: 3-hexanone, VOC 4: octanol, VOC 5: 2-ethyl-hexanol, VOC 6: tetradecane, VOC 7: hexadecenoic acid, VOC 8: dodecane. (d) Box-and-whisker plots show the distribution of abundance in concentration of value (μM) of four selected VOCs detected in exosomal fraction. (e) Schematic illustration of the preparation of VOC-encapsulated liposomes and representative image of the GCMS chromatogram of octanal-encapsulated liposomes and octanal pure standard. Data shown are as mean ± SEM.

The determination of octanal in the exosomal fraction of monocytes as opposed to that obtained in the conditioned media depicted reduced levels, which is expected, as some compounds may be lost in the course of exosome isolation. Additionally, some octanal may be present in the media and some other extracellular vesicles may be released from monocytes. An ongoing study by Orecchioni et al.^32^ established that octanal may serve as a ligand for olfactory receptor Olfr2/OR6A2, which is found in human monocyte-derived macrophages and is proinflammatory, activating the NLRP3 inflammasome and inducing IL-1β secretion. Regarding cancer cells, the ketone 3-hexanone was discovered to be more abundant in A549 as compared to KO and WT cells (Figure 3c,d, Supporting Information Table S2) (*C*_U937_ = 0.016 μM, *C*_KO+LPS_ = 0.0202 μM, *C*_KO_ = 0.015 μM, *C*_LPS+U937_ = 0.0205 μM, *C*_A549_ = 0.0402 μM). This corroborates with earlier studies that observed increased 3-hexanone in an A549-conditioned medium.^33^ When evaluating the volatile output of the exosomal fractions separated from cells, one can identify their highly lipophilic characteristics such as the highly lipophilic alkane, tetradecane, and the fatty acid, hexadecenoic acid (Figure 3c). Exosomes have been reported to encapsulate a broad selection of lipophilic materials, comprising lipids, lipid-soluble molecules, and hydrophobic substances such as fat-soluble vitamins (e.g., vitamins A–K), lipid-soluble signaling molecules (e.g., prostaglandins and leukotrienes), and other lipophilic compounds. These molecules can be situated in the lipid bilayer or attached to the hydrophobic regions of exosomal proteins.^33^

### VOC-Encapsulated Liposomes Modulate the NF-κB Pathway in Monocytes

To understand the effect of exosomes as delivery systems for VOCs, we sought to investigate this phenomenon in vitro. Our approach entailed utilizing liposomes, a lipid-based nanocarrier that is biocompatible and biodegradable, to mimic exosomes’ characteristics.

Five VOCs, 2-butanone, octanal, octanol, 1-butanol, and 3-hexanone, which showed significant variations in earlier experiments, were selected for study in NFKB1 knockout and naïve cells. Extrusion of the VOC encapsulation was done in order to procure a consistent-sized mixture of liposomes (100–120 nm), which is specified in Table S3 alongside other physical–chemical properties. To calculate the encapsulation efficiency (EE %), GCMS was employed and compared to standard curves of pure compounds (Figure 3e). EE was calculated by the following equation:

$$EE\;\left(\%\right):\frac{C\left(total\right)-C\left(free\right)}{CC\left(total\right)}$$

where *C*(total) is the concentration of the applied volatile and *C*(free) is the amount of free volatile measured in the headspace. The calculated EE % shows that octanol, 3-hexanone, and octanal have higher values with 94, 93%, and 92%, respectively, compared to 2-butanone and 1-butanol, which reached 89% and 86%, respectively. This difference in EE % may be attributed to the volatile interaction with the hydrophobic lipid bilayer.

The U937 cells were incubated with the fabricated liposomes for 10 h in order to ascertain the cellular uptake, in which case the Rhodamine-labeled phospholipids were present in the cytoplasm of the cell.

For our investigation into the effect of volatile-encapsulated liposomes on NF-κB activation, we conducted an in vitro experiment in which the liposomes were incubated with monocytes in both the presence and absence of LPS. By exposing the monocytes to the liposomes, we attempted to determine the potential modulation of NF-κB activation due to the presence of the encapsulated VOCs. We sought to comprehend the effects of volatile encapsulated liposomes on NF-κB-mediated immune responses in monocytes and thus included LPS in certain experimental conditions to assess the synergistic or additive effects. The incubation was done with the liposomes (2.5 μM) for a duration of 10 h (*n* = 4). After incubation, a multiplex analysis approach was performed to evaluate the NF-κB-related proteins (NF-κB, TNFR1, c-MYC, FADD, IkBa, and IKKα/β).

In line with previous findings, we noted that LPS stimulation caused a considerable increase in the levels of phosphorylation of NF-κB and IKKα/β in naïve and KO cells (Figure 4). Even though there was no significant difference in NF-κB-associated proteins in unstimulated cells upon incubation with 2-butanone-encapsulated liposomes, the addition of LPS to U937 cells brought about a substantial decrease in phosphorylated NF-κB and IKKα/β (Figure 4a). Additionally, we found statistically significant decreases in IkBa and TNFR1 following the addition of 2-butanone-encapsulated liposomes to LPS-stimulated cells (Figure 4a). This indicates that the liposomes inhibited the NF-κB signaling pathway, as a reduction of phosphorylated NF-κB in LPS-stimulated U937 was seen after the incubation with empty liposomes. Further, the influence of two alcohols, namely, 1-octanol and 1-butanol, was studied. Elevated levels of both these compounds were detected in KO and LPS-stimulated U937 (Figure 2e,f). Contrary to 2-butanone-encapsulated liposomes, octanol and butanol had a noteworthy effect on the expression of proteins downstream of NFκB (total c-Myc protein and phosphorylated FADD at Ser194). An increase in the expression of c-Myc protein in LPS-stimulated U937 by 1.3 and 1.5 folds was seen after incubation with 1-butanol- and octanol-encapsulated liposomes, respectively (Figure 4b,d). Additionally, FADD levels in LPS-stimulated U937 were increased by 1.2-fold for 1-butanol liposomes and 1.3-fold for octanol-encapsulated liposomes (Figure 4b–d). In naïve U937 and LPS-stimulated U937, the upregulation of *p*-IκBα, owing to the presence of 2-butanol and octanol liposomes, indicates its dissociation from the NFκB dimer, enabling it to enter the nucleus and initiate transcriptional activation, while *p*-IκBα is focused on by ubiquitin ligase proteins, causing its proteasomal degradation (Oeckinghaus and Ghosh, 2009). Thus, our experiment illustrated the capacity of volatile-encapsulated liposomes to modulate NF-κB-mediated immune responses in monocytes.

**Figure 4:**
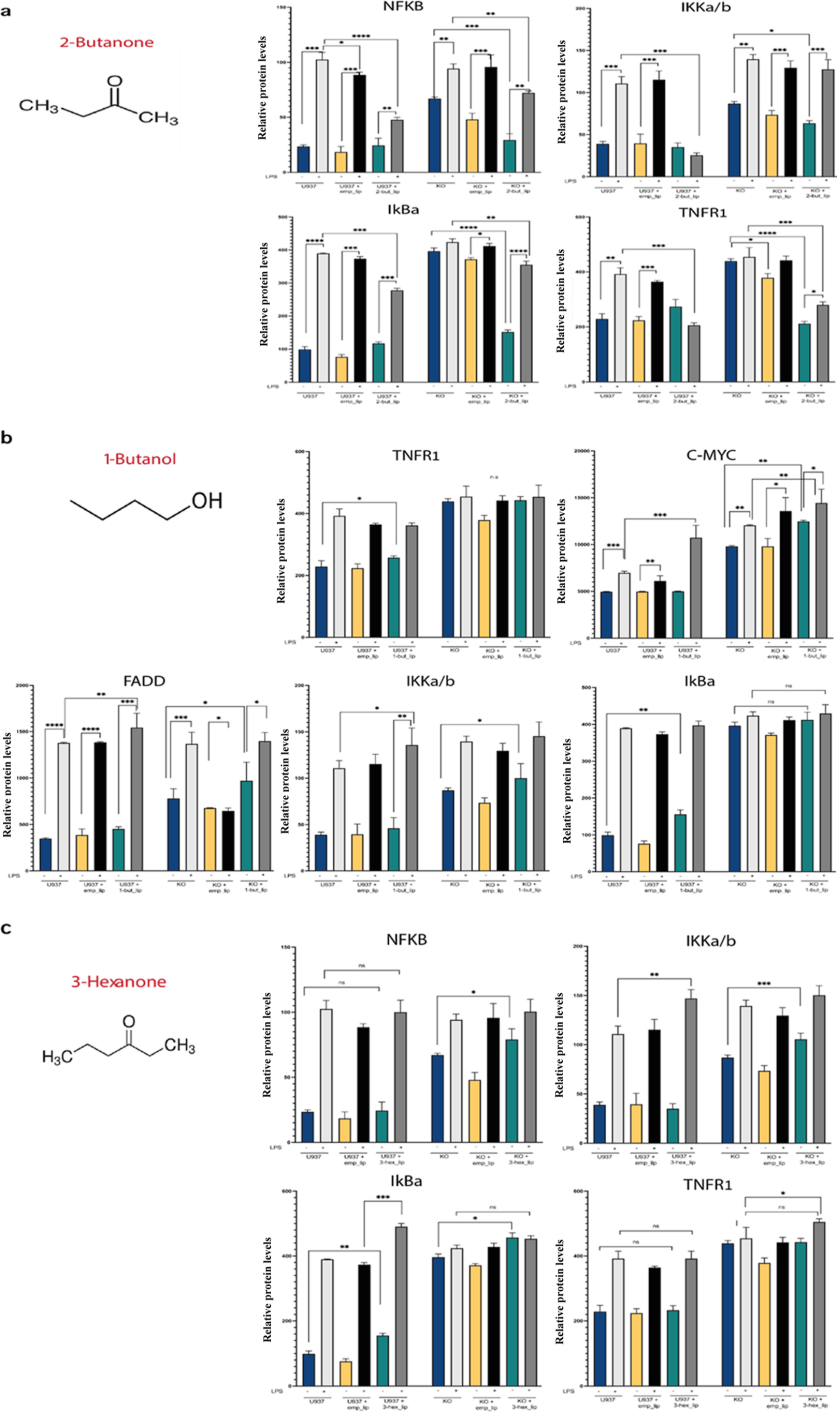

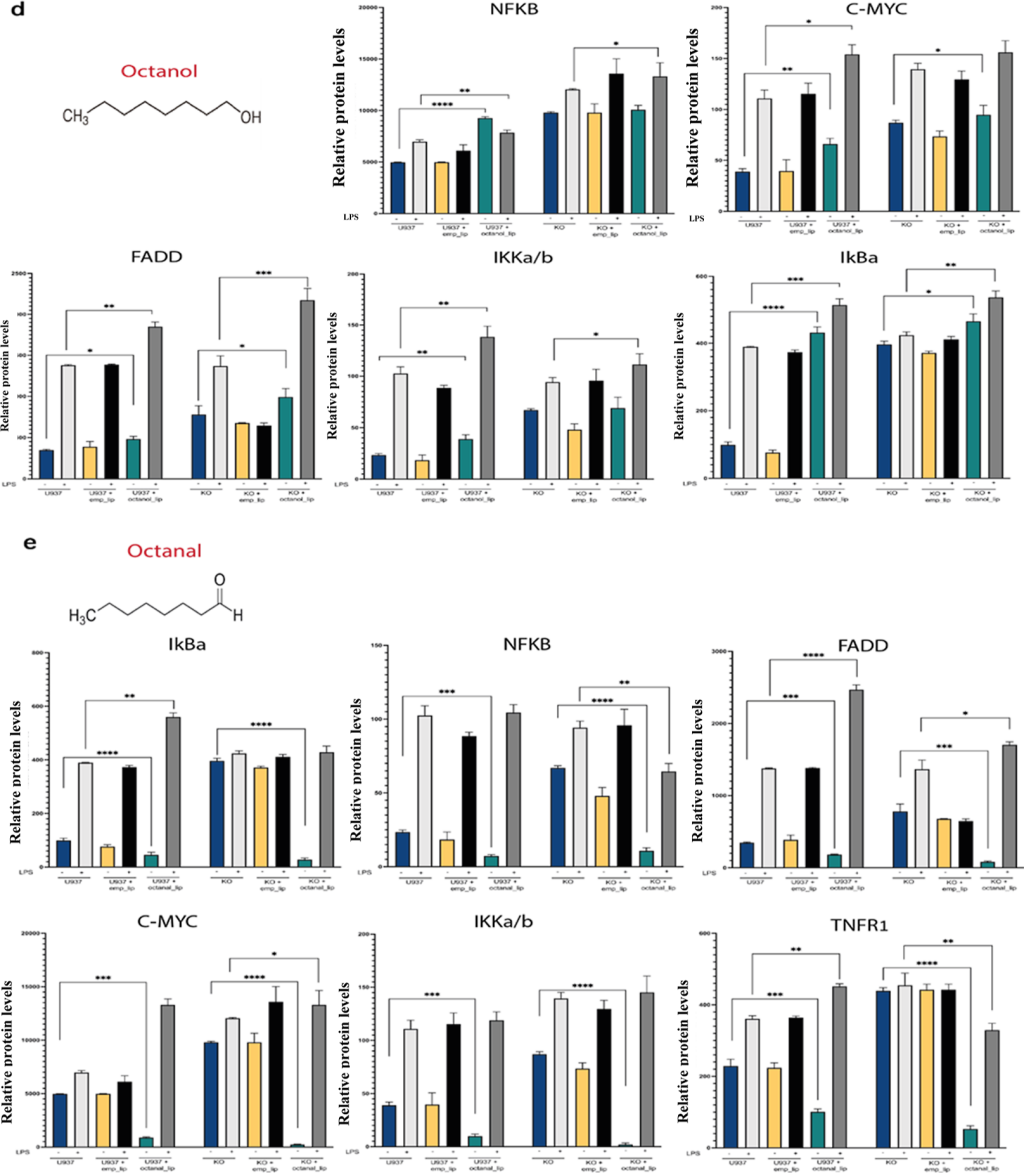
VOC-encapsulated liposomes modulate NF-κB signaling in U937 monocytes. Relative protein abundance and phosphorylation levels of TNFR1, *p*-FADD (Ser194), *p*-NF-κB (Ser536), *p*-IκBα (Ser32), and total c-Myc in wild-type (WT; naïve U937) and NFKB1-deficient U937 (KO) cells following incubation with liposomes encapsulating (a) 2-butanone, (b) 1-butanol, (c) 3-hexanone, (d) octanol, or (e) octanal. Data are presented as mean ± SEM (*n* = 4). Statistical significance was determined using Student’s *t*-test (*p* < 0.05).

Regarding the effects of encapsulated alcohols in KO cells, we find a pattern comparable to that of U937 cells. Butanol-encapsulated liposomes generated a higher presence of downstream proteins FADD and c-Myc in both naïve and LPS-stimulated KO cells, while IKKα/β only showed an increase in the naïve KO cells (Figure 4b). NFκB, IkBa, and TNFR1 showed no marked shift when the butanol-encapsulated liposomes were administered (Figure 4b). With respect to octanol-encapsulated liposomes, an increase of NFκB and its regulators (IkBa and IKKα/β), as well as the proteins downstream of NFκB (overall c-Myc protein and phosphorylated FADD at Ser194), was noticed in both naïve and LPS-stimulated U937 cells (Figure 4d). On the other hand, a noteworthy elevation in *p*-FADD and *p*-IkBa was detected in both naïve and LPS-stimulated KO cells, with c-Myc increasing only in naïve KO cells and *p*-IKKα/β increasing in LPS-stimulated KO cells (Figure 4d).

In a previous study, the aldehyde octanal was included in liposomes and found to be important in noncontact communication between lung cancer cells, normal cells, and monocytes (Hashoul et al., 2023). It was likewise established that octanal was high in KO cells and LPS-stimulated U937 in both the conditioned medium and liposomal fraction (Figures 2e,f and 3d,e). Noteworthily, octanal decreased the levels of all tested NFκB proteins (TNFR1, *p*-FADD (S194), *p*-NFκB (S536), *p*-IKKα/β (Ser177/Ser181), *p*-IκBα (S32), and c-Myc). At the same time, it also improved the phosphorylation levels of IkBa, FADD, and total amounts of c-Myc and TNFR1 (Figure 4e). Similar to the observations in KO cells, a remarkable decline was detected in all NFκB proteins. A rise in *p*-NFκB and TNFR1 was detected, apart from the overall c-Myc and *p*-FADD levels, which both were seen to be increased in LPS-stimulated KO cells (Figure 4e). In consideration of the presented information, resveratrol appears to be a possible suitable choice. This naturally occurring polyphenol is present in plants such as grapes and berries and is known to have anti-inflammatory properties. Its utilization has been studied to curb NF-κB overactivation, which leads to heightened inflammation and thus furthers inflammatory illnesses. Studies have displayed resveratrol’s ability to inhibit NF-κB activation and reduce the inflammatory response in such conditions.^34^ Yet, resveratrol has also been seen to be counterproductive when incorporated with LPS in that it induces the generation of proinflammatory cytokines.^35^

### 2-Butanone- and Octanal-Encapsulated Liposomes Alter the Expression of Several Inflammatory Cytokines

Due to the noted change in NF-κB activation, we decided to evaluate further the effects of 2-butanone- and octanal-encapsulated liposomes on the secretion of various inflammatory cytokines (IL-10, MCP-1, TNF-α, IL-1β, IL-6, and IL-8). The cells were incubated with the liposomes (2.5 μM, *n* = 4) for 10 h, and the supernatant was then analyzed for cytokine levels. Results revealed that incubation with empty liposomes resulted in an anti-inflammatory effect which is clearly illustrated by the reduction of IL-1β, IL-8, and TNF-α levels (Figure 5a). Moreover, 2-butanone-encapsulated liposomes caused a decrease in the proinflammatory cytokines (IL-1β, IL-6, IL-8, and TNF-α) in both naïve U937 monocytes and LPS-stimulated U937 monocytes (Figure 5a). When 2-butanone-encapsulated liposomes were incubated with unstimulated U937, the level of IL-10 was notably elevated; however, LPS-stimulated U937 showed no substantial variation (Figure 5a). A similar pattern was observed in KO cells as well; 2-butanone-encapsulated liposomes significantly reduced the levels of IL-1β, IL-6, IL-8, MCP-1, and TNF-α in both KO and LPS-stimulated KO cells (Figure 5a). With regard to IL-10, there was no remarkable difference in KO cells, while a decline was detected in LPS-stimulated KO cells (Figure 5a). It should be kept in mind that during the early stages of LPS stimulation, there might be an increase in IL-10 production by monocytes as a regulatory measure to restrict extreme inflammation.^36^ Nonetheless, as the immune response intensifies and inflammatory signaling pathways become more active, the IL-10 levels could possibly diminish. This decrease might be caused by the dominance of proinflammatory cytokines and the progression to a more inflammatory state.^36,37^ On the other hand, octanal-encapsulated liposomes noticeably raised IL-1β, IL-6, IL-8, MCP-1, and IL-10 in LPS-stimulated U937, which indicates an inflammatory reaction (Figure 5b).

**Figure 5:**
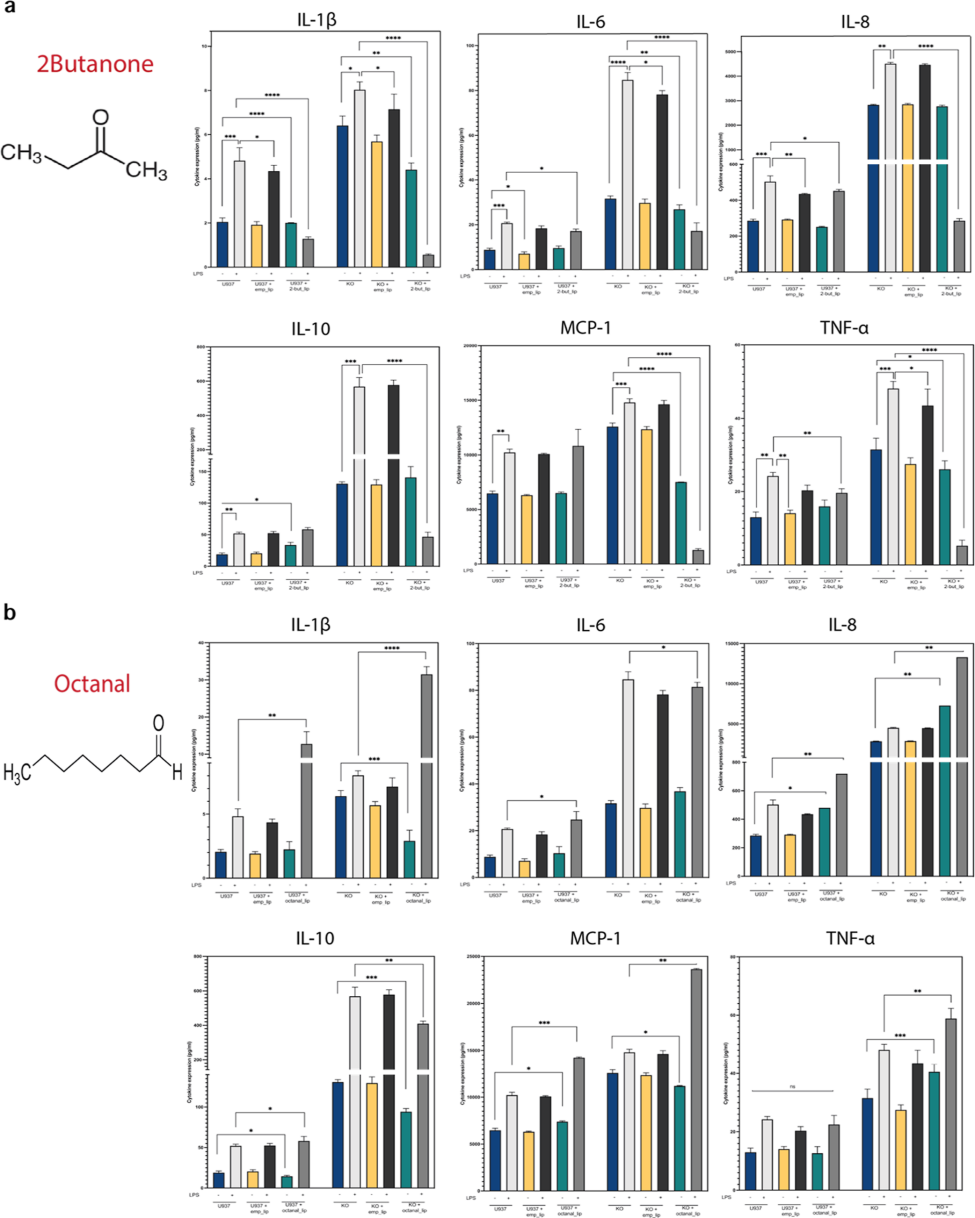
VOC-encapsulated liposomes modulate cytokine secretion in U937 monocytes. Cytokine levels of IL-10, MCP-1, TNF-α, IL-1β, IL-6, and IL-8 in wild-type (WT; naïve U937) and NFKB1-deficient U937 (KO) cells following incubation with liposomes encapsulating (a) 2-butanone or (b) octanal. Data are presented as mean ± SEM (*n* = 4). Statistical significance was determined using Student’s *t*-test (*p* < 0.05).

Regarding the effect of octanal-encapsulated liposomes in KO cells, an opposite pattern was observed between KO and LPS-stimulated KO cells. A decrease in IL-1β, IL-10, and MCP-1 was evident in KO cells, while IL-8 and TNF-α levels were elevated (Figure 5b). In LPS-stimulated KO cells, octanal-encapsulated liposomes significantly increased IL-1β, IL-8, MCP-1, and TNF-α, while decreasing IL-6 and IL-10.

## Discussion

NF-κB is a critical transcription factor in monocytes and macrophages, regulating their versatile functions in immune responses and inflammation. Its activation and coordination of transcriptional and epigenomic programs play vital roles in monocyte differentiation, macrophage polarization, inflammasome activation, and cell survival. Here, we explored how VOCs affected the NF-κB activation in the monocytic cell line (U937) and NFKB1-knockout U937 (KO) human monocyte cells. Results show a noticeable increase in NF-κB and its regulators (IKKa/b and IkBa) after 1 day of culturing, followed by a progressive decline that lasts until the third day of culturing. This NF-κB change is evident throughout the four-day culturing period. These NF-κB fluctuations across the culturing period may indicate feedback mechanisms for the concentration of volatile molecules emitted from cancer cells. NF-κB activation is subject to complex regulatory mechanisms involving feedback loops and interactions with other signaling pathways, and fluctuations in its activity may arise from the interplay between different signaling pathways, cellular conditions, and feedback regulations.^38^

To further explore the NF-κB pathway and its associated volatile signature, we employed CRISPR/Cas9 gene editing approaches to generate a NFKB1-knockout U937 (KO) human monocyte cell. NFKB1 encodes the p105 subunit of NF-κB, which is processed to create the NF-κB p50 subunit. NFKB1 is the most abundantly expressed transcription factor in macrophages and is a key initiator of chronic inflammatory disease. In addition to regulating TLR-induced proinflammatory gene expression in activated macrophages, NFKB1 also plays a key role in shaping macrophage polarization and innate immune memory responses.^14^

The divergent responses to VOC exposure observed between wild-type and NFKB1-deficient monocytes can be interpreted in the context of altered NF-κB dimer composition, resulting from p50 deficiency. In wild-type cells, NF-κB signaling is predominantly mediated by p65/p50 heterodimers, which typically regulate a balanced transcriptional program encompassing both proinflammatory and regulatory target genes. In contrast, the loss of p50 shifts the equilibrium toward alternative NF-κB dimers, including p65 homodimers, which have distinct DNA-binding affinities, transcriptional activities, and regulatory outcomes. This altered dimer composition is predicted to reprogram transcriptional responses downstream of NF-κB activation, leading to context-dependent differences in the cytokine expression and signaling outputs.

Within this framework, VOCs such as 2-butanone and octanal may differentially modulate NF-κB-dependent transcription, depending on the availability of specific NF-κB dimers. For example, VOC-induced suppression of NF-κB signaling in wild-type cells may reflect inhibition of canonical p65/p50-mediated transcription, whereas in NFKB1-deficient cells, the same VOCs may engage alternative NF-κB complexes or compensatory pathways, resulting in divergent or even opposing transcriptional outcomes. Together, these observations suggest that the p50-dependent NF-κB dimer composition serves as a key determinant of VOC-specific transcriptional responses in monocytes.

GCMS analysis revealed a unique volatile signature for KO when compared to naïve and LPS-stimulated U937; for instance, decreased levels of 2-butanone and propanol and increased levels of octanal and heptanone were observed in LPS-stimulated cells when compared with WT cells. These results can provide insights into the underlying metabolic and biochemical processes of immune responses. Also, integrating GCMS data with other omics data, such as genomics, proteomics, and metabolomics, allows for a more comprehensive understanding of the complex mechanisms underlying inflammation.

Given the lipophilic nature of VOCs, we hypothesized that VOCs, like other signaling molecules, utilize exosomes as carriers. Hence, they can overcome their inherent limitations and effectively circulate in the bloodstream, expanding their reach and potential impact on various biological processes. The rationale behind the above hypothesis stems from numerous reasons: first, exosomes can provide protection and prevent VOC degradation. Second, exosomes are known to traffic between cells and tissues, offering a potential mechanism for VOCs to be transported to distant sites in the body. Additionally, the ability of exosomes to cross biological barriers, such as the blood–brain barrier, further enhances their potential for facilitating VOC distribution to target tissues. Lastly, exosomes are involved in systemic communication, making them an ideal vehicle for VOCs to be delivered to specific cell types or organs, exerting their physiological or pathological effects.^39–41^

We separated the exosomal fraction from the lung cancer cell line (A549) and KO/U937 cells and examined their volatile production with ITEX-GCMS. We successfully displayed a distinct volatile signature for the tested cell. However, the current experimental design did not include a quantitative comparison between vesicle-associated VOCs and those present in the bulk conditioned medium and, therefore, does not allow conclusions regarding preferential enrichment or transport efficiency. Importantly, the data do not distinguish between active, regulated vesicular loading and passive incorporation driven by physicochemical partitioning into membrane lipids during vesicle biogenesis or isolation. Additionally, alternative carriers, including other lipid-containing extracellular components, cannot be excluded. Thus, while the detection of VOCs in exosomes supports the plausibility of vesicle-mediated VOC transport, this mechanism remains speculative and will require direct quantitative and mechanistic validation. Next, we encapsulated selected VOCs inside liposomes to study their impact on NF-κB activation. The rationale for encapsulating VOCs in liposomes for in vitro experimentation and potential future in vivo investigations lies in harnessing the benefits of liposomal delivery to explore their functional impact and potential translational applications, especially in lung diseases. While we did not directly measure VOC retention during incubation, encapsulation within liposomal carriers is an established strategy to moderate volatility and improve persistence of active compounds in aqueous and biological environments. Liposomal and related nanocarrier systems have been applied in agricultural contexts for stabilizing volatile bioactives and improving delivery efficiency under experimental conditions analogous to those used here.^48^

2-Butanone-encapsulated liposomes showed an evident decrease of four associated NF-κB proteins (TNFR1, *p*-NFκB (S536), *p*-IKKα/β (Ser177/Ser181), and *p*-IκBα (S32)), along with a decrease in levels of IL-1β, IL-6, IL-8, MCP-1, and TNF-α in both KO and LPS-stimulated KO cells. Alternatively, there is no significant change in *p*-FADD and total c-Myc. The absence of changes in downstream genes such as c-Myc and FADD may be due to several factors. First, the kinetics of gene expression can vary, with some genes responding rapidly to NF-κB activation, while others exhibit delayed or sustained responses. It is possible that the time point at which gene expression was assessed in the study did not capture changes in the c-Myc and FADD expression. Second, NF-κB is a pleiotropic transcription factor that regulates various genes with diverse functions. While NF-κB directly regulates some downstream genes, others may require additional cofactors or transcriptional coregulators to activate. The differential regulation of downstream genes could be influenced by the presence or absence of these cofactors or coregulators, which may vary in different experimental settings.

Furthermore, the post-translational modifications and interactions of NF-κB with other transcription factors and coregulators can modulate its transcriptional activity and specificity. These complex regulatory mechanisms can activate specific sets of target genes in a context-dependent manner. Overall, the observed increase in phosphorylated NF-κB and IKKα/β, as well as IkBa, without changes in downstream genes such as c-Myc and FADD, suggests that the NF-κB signaling pathway may be activated. Still, its downstream effects on specific target genes might be regulated through additional layers of complexity, such as temporal dynamics, cofactors, and post-translational modifications. Further investigations are needed to unravel the precise mechanisms underlying these observations and to understand the full spectrum of NF-κB-mediated gene regulation.

Octanal-encapsulated liposomes showed inverse effects on NF-κB activation, in which, under basal conditions, liposomes decreased the level of NF-κB activation by reducing the levels of all NF-κB associated proteins in U937 WT and KO cells. In addition, cytokine analysis revealed a decrease in the levels of IL-1β, IL-10, and MCP-1 in KO cells, while IL-8 and TNF-α levels were elevated. Alternatively, upon the addition of LPS, an opposite effect manifests in which an increase in *p*-IkBa, *p*-FADD, c-Myc, and TNR1 was evident in LPS-stimulated cells. This NF-κB activation alteration was accompanied by an increase in IL-1β, IL-8, MCP-1, and TNF-α levels while decreasing IL-6 and IL-10 in LPS-stimulated cells. These inverse effects observed in KO cells may arise from impaired NF-κB response since LPS stimulation triggers the activation of NF-κB in immune cells.^42^ In NFkB1 knockdown cells, the availability of p50 subunits derived from the p105 precursor is reduced. Consequently, the ability to form active NF-κB complexes and mount a robust NF-κB response to LPS stimulation can be impaired.^42,43^ Indeed, NFkB1 knockdown has been shown to have inverse effects on NF-κ B activation in various cellular contexts. In siRNA-mediated knockdown models of the RelA and NFkB1 genes, IL-6 decreased miR-24 expression via the NFkB-mediated pathway.^42^ Additionally, the knockdown of NFkB1 has been shown to reduce inflammation in various central nervous system injury models.^43^ Knockdown of NFkB1 has also been shown to sensitize cervical cancer cells to doxorubicin-induced cell toxicity.^44^ Moreover, this differential cytokine response upon liposomal octanal exposure in WT monocytes compared to NFKB1 KO cells can be attributed to the distinct roles of NF-κB subunits in redox-sensitive transcriptional regulation. Octanal, an aldehyde derived from lipid peroxidation (LDA), is produced during oxidative stress and has the capacity to influence cellular signaling pathways. In WT monocytes, octanal activated the canonical NF-κB pathway, leading to the phosphorylation of IκBα, nuclear translocation of p65/p50 heterodimers, and subsequent transcription of genes involved in inflammation and cell survival. This activation is facilitated by the redox-sensitive nature of NF-κB, where reactive oxygen species (ROS) serve as secondary messengers in signal transduction. Conversely, P50-deficient monocytes exhibit a modified response to encapsulated octanal. The lack of the p50 subunit hinders the formation of p65/p50 heterodimers, leading to a predominance of p65 homodimers or other alternative complexes. These alternative dimers possess varying DNA-binding affinities and transcriptional activities, which may cause the dysregulation of NF-κB target genes. Additionally, the absence of p50 disrupts the negative feedback mechanisms that typically govern NF-κB activation, potentially resulting in prolonged or uncontrolled inflammatory responses. Moreover, LDAs such as octanal can create adducts with proteins, thereby impacting their functionality and further influencing signaling pathways. In p50-deficient cells, the altered redox balance and compromised NF-κB signaling may intensify the cytotoxic effects of octanal, leading to heightened cellular stress and apoptosis.^47–49^

To conclude, we report, to the best of our knowledge, the first evidence that exosomes isolated from various cells (U937 and A549) display distinct volatile output as well as within the same cells both at rest and when LPS is triggered (U937). Moreover, we demonstrated a unique volatile signature for NFKB1 KO cells. We thoroughly investigated the impact of two volatiles contained within liposomes on the NF-κB pathway and the release of numerous proinflammatory outcomes. According to our findings, 2-butanone-encapsulated liposomes can significantly reduce NF-κB activation and limit the expression of proinflammatory cytokines in LPS-stimulated cells (KO/U937). Conversely, octanal-encapsulated liposomes showed inverse effects in KO and naïve cells, which indicated a complex and context-dependent effect on the NF-κB pathway in monocytes. Specifically, octanal deactivates the NF-κB pathway in WT cells (U937/KO), reducing NF-κB activation and downstream proinflammatory cytokine expression. However, when applied to LPS-stimulated cells, octanal activates the NF-κB pathway, suggesting a potential compensatory mechanism. These findings highlight the intricate nature of the octanal’s interaction with the NF-κB pathway and its regulatory mechanisms in different cellular contexts. The compound’s ability to deactivate the NF-κB pathway in monocytes underscores its potential as an anti-inflammatory agent, providing a means to suppress excessive inflammation associated with various diseases.

Similarly, the reduction in NF-κB activation and proinflammatory cytokine expression suggests that octanal may mitigate inflammatory responses and ameliorate inflammatory conditions. Furthermore, the observation that octanal activates the NF-κB pathway in LPS-stimulated monocytes indicates its potential to bypass or interact with NFKB1-independent pathways for NF-κB activation. These findings highlight the complexity of NF-κB regulation and suggest that the compound may have broader effects on NF-κB signaling beyond its interaction with NFKB1.

Additional research is required to completely comprehend the mechanism of action of 2-butanone and octanal and any possible therapeutic applications. Thorough investigations that clarify their molecular targets, downstream signaling cascades, and potential off-target consequences will be helpful. Also, the exact mechanism by which VOCs exert their action is not well-characterized; nonetheless, several studies have demonstrated that olfactory receptors, originally characterized in the olfactory epithelium, are also expressed in nonolfactory tissues, including immune cells such as monocytes and macrophages. Functional activation of specific olfactory receptors in macrophages by volatile ligands has been shown to trigger intracellular signaling cascades and inflammatory responses, including pathways intersecting with NF-κB signaling.^49^ These observations raise the possibility that certain VOCs may modulate monocyte responses through receptor-mediated mechanisms, in addition to or in parallel with direct cellular uptake and intracellular metabolism. Given the small size and lipophilic nature of many VOCs, both modes of action are plausible and are not mutually exclusive. It will therefore be interesting in future studies to delineate the precise mechanism by which different VOCs enter or signal within monocytes, including the relative contribution of membrane receptor engagement versus intracellular metabolic processing.

Moreover, in vivo studies are necessary to evaluate the compound’s effect and suitability for therapeutic applications. Furthermore, while the findings suggest potential implications for pulmonary diseases, their physiological relevance remains to be confirmed. Future research should incorporate in vivo models to assess the biological effects of VOCs within the complex environment of the lung and to evaluate the therapeutic potential of targeting VOC-mediated communication pathways.

## Methods

### Cells

Three cell lines were used in this study: A549 (adenocarcinoma lung epithelial cells), BEAS-2B (normal human lung epithelial cells), and U937 (promonocytic human myeloid). All cell lines were purchased from the American Type Culture Collection (ATCC, Manassas, VA, USA). Cell lines were maintained in RPMI 1640 medium. In addition, 10% fetal bovine serum and 1% penicillin and streptomycin were added to the RPMI (all from Biological Industries). The cells were grown to 60–90% confluency in the 75 cm^2^ culture flask under standard conditions at 37 °C and 5% CO_2_.

### Culture Conditions

All volatile communication setups described herein were performed in separate incubators for each tested group. All incubators were sterilized by high-temperature decontamination prior to any experiment. Also, all cell lines (U937, A549, BEAS-2B) used in experiments were between passage 3 and 5 (U937: 3, A549: 5, BEAS-2B: 4) while each cell line passage was consistent through the different setups.

### Headspace Sampling of the One-Way Communication Setup

Microfluidic devices were custom designed and fabricated by FEMTOprint glass-3D printing technology (FEMTOprint, Switzerland). The microfluidic chamber incorporates a Tesla-type passive valve that enables preferential unidirectional gas-phase transport through an asymmetric channel geometry, resulting in substantially higher flow resistance in the reverse direction. This design minimizes back-diffusion and promotes directional transmission of gaseous VOCs without the need for active pumping or imposed pressure gradients. The principle, fabrication, and experimental validation of this Tesla valve-based communication platform, including characterization of directional flow behavior and VOC transmission, have been described in detail in our previous work.^12^ All devices were autoclaved prior to use, and all cell combinations and controls (A → U, M → U, M → M, A → M) were prepared as follows: all three cell lines were grown to a 60–90% confluency and were washed with phosphate buffer solution (PBS) and detached with 0.25% EDTA (A549). The number of seeded cells in microfluidic devices was determined based on optimization experiments aimed to assess the proliferative rate of each cell line and any stress resulting from the culturing. Kima pumps (Cellix Ltd.) were used for media perfusion (10 μL/min) to circulate media from a 5 mL medium reservoir. All microfluidic chambers (*n* = 6 per group) were cultured in separate incubators for each group for 4 days. At the end of the incubation period, cell number and viability were examined, respectively, by cell count and Trypan-blue. Headspace VOCs were actively sampled and preconcentrated on Tenax TA sorbent tubes at defined time points (day 1 and day 4) using a disc pump (ttpventus). Sampling was performed at a controlled flow rate of 10 mL/min for a fixed collection duration per sample, as specified by the pump settings, to ensure reproducible VOC capture across experimental conditions. The tubes were run in thermal desorption (TD) followed by GC-MS analysis. Blank control measurements were performed using identical microfluidic chambers containing culture medium only (no cells), sampled under the same incubation, flow, and headspace collection conditions as experimental samples.

### Gas Chromatography–Mass Spectrometry (GC-MS) Analysis

GC-MS analyses were performed using an Agilent 7890B series GC system (Agilent, USA) connected to an Agilent 5977A mass selective detector (MSD) (Agilent, USA) equipped with an extractor EI source. The analytical column was a SLB-5 ms capillary column (with 5% phenyl methyl siloxane; 30 m in length; 0.25 mm in internal diameter; 1 μm in thickness; from Sigma-Aldrich). For exosome sampling, ultrahigh-purity (99.999%) helium was used as carrier gas (flow rate 1 mL/min). The GC was operated under the following temperature program: initially at 35 °C, held for 10 min at 200 °C, held at 240 °C for 21 min, ramped at 15 °C min^–1^ to 260 °C, and held at 260 °C for 2 min, giving a total run time of 25.7 min.

### In-Tube Extraction (ITEX) Method

For exosome sampling, we used an ITEX connected to a GC-MS system (CTC Analytics AG, Switzerland). The extraction process is fully automated and performed dynamically by moving the plunger of the syringe up and down to pump the sample headspace through the sorbent bed. Then, a fixed volume of an inert gas is aspirated into the syringe as the desorption volume. Before the desorption process, the external heater is rapidly heated to the desorption temperature and the analytes are ejected into the GC injector. After the needle is withdrawn from the GC injection port, the extraction device is flushed with an inert gas and heated to prevent carryover and to condition the extraction for a sample.^45,46^ The sample vial was set on an automatic sampling system connected to the GC-MS instrument (Auto-PAL-RSI 120 system). Automated ITEX applied a 1.3 mL headspace syringe with a Tenax TA-filled needle body. The analytes were extracted from the sample headspace by dynamic extraction onto the absorbent. The autosampler was equipped with a single magnet mixer (SMM) and a temperature-controlled tray holder. The samples were placed in the tray cooler at 25 °C; after transfer to the SMM, the sample was heated and stirred at 500 rpm for 20 min to reach the extraction temperature of 80 °C to establish equilibrium distribution of the analytes between the liquid and gas phase in the vial before extraction. The extraction volume of the gas phase was set to 1000 μL, and 750 extraction strokes (20 s for each stroke) were used for the optimized method for each sample. The extraction flow rate during extraction was set at 100 μL/s. After extraction, the sample vial was moved back to the tray. Desorption was performed in step 3, the ITEX trap was heated to 250 °C with a desorption flow rate of 1 mL of purge gas was used to desorb and purge the extracted volatiles of the sample at a flow rate of 10 μL/s into the hot injector. After desorption, the ITEX device was flushed with nitrogen gas at 260 °C for 5 min. Afterward, the plunger was moved down and the temperature was set to 80 °C to prepare the trap for the next extraction. The whole process (including injection, trap cleaning, and extraction of the following sample) was completed within the runtime of the GC oven program with cooling about 5 h. All experiments were repeated five times, and the results were expressed as the mean ± standard deviation.

### GC-MS Data Processing

Compounds were tentatively identified through a spectral library match NISTL.14 (National Institute of Standards and Technology, USA). VOCs detected in blank control chambers containing the culture medium only were used to define background signals originating from the chamber materials, culture medium, or environmental sources. These background VOCs were subtracted from experimental samples prior to statistical analysis, ensuring that the reported signals reflected cell-derived VOCs. The Kruskal–Wallis test and an extension of the nonparametric Wilcoxon test, including Bonferroni alpha correction, were used to identify significantly altered VOCs. SAS JMP, version.14.0 (SAS Institute, Cary, NC, USA; 1989, 2005), was used for statistical analysis.

### Quantitative Analysis

Chemicals and Standards. All calibration mixtures were made using high-purity liquid substances and a protocol outlined in detail elsewhere; 57, briefly, reference chemicals with stated purities of 95–99.9% were purchased from Sigma-Aldrich (MI, USA) and were used without any further purification. Volatile identification and concentration were determined through external standards and calibration curves. For each volatile, the reagents and stock solutions were made to a concentration of 1 M by dissolving them in 1 mL of methanol. Calibration solutions of 2–500 μg/L were prepared in methanol. Volatile standards were diluted and measured by using the same methods for measuring samples. Standard curves were created based on the peak areas, which were obtained from Mass Hunter Qualitative analysis. The data were analyzed in triplicates. Compound identification was performed using a two-step process. First, the spectrum of a peak was checked against the NIST mass spectral library database. Next, the NIST identification was confirmed by comparing the retention times of peaks of interest with the retention times obtained for reference standards prepared as outlined above.

Quantification of liposome-encapsulated volatiles. The quantification of the volatiles released from liposomes was performed by using the ITEX method. The solutions of liposome samples were centrifuged at 9000 rpm for 10 min. Subsequently, the liposome fractions were recovered, and an aliquot of 600 μL was dissolved in 1 mL of methanol and sampled by the headspace. To determine the encapsulation efficiency (EE), the previously described method was used to measure the concentration of pure standards. EE was calculated by the following equation: EE (%): (*C*(total) – *C*(free))/(*C*(total)), where *C*(total) is the concentration of applied volatile and *C*(free) is the amount of free volatile measured in the headspace.

### CRISPR/Cas9 Mediated Knockout

To generate a NFKB1 knockout in U937 cells, a pair of guides RNAs targeting exon 1 of NFKB1 were chosen from the article^39^ (spacer sgRNA#1: CAGGTAGTCCACCATGGGAT, spacer sgRNA#2: GAACAAGAAGTCTTACCCTC). Spacers were cloned into lentiCRISPR v2 plasmid (Addgene plasmid # 52961) restricted by BsmBI restriction enzyme (NEB #R0580, BioLabs) and ligated by T4 DNA ligase (#M0202L, BioLabs) together with nontargeted negative control (empty lentiCRISPR v2 plasmid). Next U937 cells were cotransduced using lentivirus particles with both sgRNAs according to PolyJet In Vitro DNA Transfection Reagent protocol (no. SL100688, SignaGen laboratories). Five days following transduction, genomic DNA was extract from cells U937 using the Quick-DNA miniprep plus kit (#D4068, Zymo), and the area around the protospacer was amplified by PCR using Taq DNA polymerase (#M0273, BioLabs) using primers: Forward: TGGCAGCAGCAATTTAAGACAAG, reverse: GGGTACTTTCAGGCTCTCTATGG) (IDT). Next, PCR products were cleaned on silica columns according to the manufacture protocol (#740609.250, Macherey-nagel). The cleaned PCR product was set to Sanger sequencing (Macrogene Europe), and the efficiency of mutagenesis on the bulk of cells was analyzed using the Inference of CRISPR Edits (ICE) (Synthego). In selected clones, which exhibited the desired genetic defect, the gene knockout was validated at the protein level by Western blot.^39^

### Western Blot for NFKB1 Knockout Validation

Whole cell lysates were generated from cells suspended in RIPA buffer containing 20 mM *Tris*–HCl (pH 7.5), 1% Nonidet P-40, 1% sodium deoxycholate, 150 mM NaCl, 1 mM EDTA, 1 mM PMSF, 1 mM EGTA, 2.5 mM sodium pyrophosphate, 1 μg/mL leupeptin, 1 mM β-glycerophosphate, and 1 mM Na3VO4. After an additional 20 min on ice, cell extracts were centrifuged for 15 min at 14,000*g* at 4 °C and supernatants. The protein concentration of lysates was determined using a Bicinchoninic Acid assay (BCA assay). Denatured samples were subjected to SDS-PAGE, transferred to PVDF membranes, and blocked for 1 h at room temperature using 5% fat free milk powder in PBS–Tween20.

Membranes were immunoblotted with specific antibodies. p105/p50 (#12540) were purchased from Cell Signaling Technologies and GAPDH (#EPR6256) was purchased from Abcam, followed by incubation with monoclonal goat antirabbit IgG H&L (Abcam) antibody (ab6721).^14^ **Liposome preparation.** A lipid mixture of DPPC (Lipoid—Ludwigshafen, Germany), cholesterol (Sigma—Rehovot, Israel), and DSPE-PEG2000 (Lipoid—Ludwigshafen, Germany) was dissolved in absolute ethanol (Bio Lab, Jerusalem, Israel) in 10% (v/v) solution volume and heated at 55 °C. 1% DPPE-Rhodamine was added to the lipid mixtures, and 1 mg/mL of each volatile was added to the lipid mixture. The lipid solution was added to the PBS buffer solution. The liposomes were downsized using a Lipex extruder (Northern Lipids, Vancouver, Canada) five times through each 400, 200, and 100 nm polycarbonate membrane (Whatman, Newton, MA, USA) at 55 °C with a maximal nitrogen pressure of 30 bar. The liposomes were dialyzed using a 12–14 kDa cutoff membrane (Spectrum Laboratories, CA, USA) against a 4 °C 5% PBS buffer solution. The external PBS buffer was replaced after 1, 4, and 24 h. Vesicle size, polydispersity index of liposomes was determined by dynamic light scattering (DLS) and stored at 4 °C. To obtain liposomes with a final lipid composition of 55% mol (HSPC/DMPC, Lipoid (Ludwigshafen, Germany)), 5 mol % polyethylene glycol distearoyl-phosphoethanolamine (m2000 PEG DSPE, Lipoid (Ludwigshafen, Germany), and 40 mol % cholesterol (Sigma (Rehovot, Israel)), accurately weighed amounts of lipids were dissolved in 10%v/v absolute ethanol (Bio Lab, Jerusalem, Israel) and warmed to 65 °C. Once all the lipids are dissolved entirely in ethanol, the lipid suspension is added to a 65 °C-heated 0.9%w/v NaCl buffer to form MLV. To obtain homogeneous nanoparticles, the mixture of lipids has to pass stepwise extrusion through polycarbonate membranes (GE Osmonics, USA), using 400, 200, 100, and 80 nm pore-size membranes in the extruder supplied with a warm bath (Northern Lipids, Vancouver, Canada). The liposomes solution is dialyzed against NaCl buffer using a 12–14 kDa dialysis membrane. NaCl buffer solution is replaced every 1, 4, and 24 h until the nonentrapped molecules are removed.

Liposomal encapsulation and similar nanoparticle platforms have been widely used to improve the stability and controlled release of volatile and semivolatile compounds in biological systems and agricultural applications, making them a rational choice for delivering VOCs in vitro. Nanocarrier encapsulation has been shown to enhance stability and biological activity of plant VOCs and protect volatile bioactive compounds from rapid degradation relative to free forms.^48^

### Liposome Treatment

U937 cells (*n* = 4) were plated at a density of 1.5 × 10^6^ cells in a 6-well plate (Thermo Fisher) in RPMI 1640 medium and stimulated with the different liposomes (2.5 μM, 10 h). In some experiments, as indicated, monocytes were pretreated with lipopolysaccharide (LPS, 100 ng/mL) for 10 h before exposure to the different liposomes.

### Cryo-TEM Liposomes Imaging

Liposomes were imaged by using cryogenic transmission electron microscopy (Cryo-TEM). Cryo-TEM imaging was performed on a Thermo-Fisher Talos F200C, FEG-equipped high resolution-TEM, operated at 200 kV. Specimens were transferred into a Gatan 626.6 cryo-holder and equilibrated below −170 °C. Micrographs were recorded by a Thermo-Fisher Falcon III direct detector camera, at a 4k × 4k resolution. Specimens were examined in TEM nanoprobe mode using Volta phase plates for contrast enhancement. Imaging was performed in a low dose mode to minimize exposure of the imaged area to electrons. Images were acquired using TEM Imaging and Acquisition (TIA) software. Liposomes were diluted 20× with saline. Cryo-TEM specimens were prepared in a controlled environment vitrification system (CEVS). Since the system under study is aqueous, preparation was done in a temperature-controlled chamber with humidity at saturation to prevent evaporation of volatiles. Temperature was kept constant at 25 °C. A drop of the solution was placed on a carbon-coated perforated polymer film, supported on a 200 nm mesh TEM grid, mounted on tweezers. The drop was turned into a thin film (preferably less than 300 nm) by blotting away excess solution with a metal strip covered with filter paper. The grid was then quickly plunged into liquid ethane at its freezing point (−183 °C). Prior to specimen preparation, grids were plasma etched in a PELCO EasiGlow glow-discharger (Ted Pella Inc., Redding, CA) to increase their hydrophilicity.

### Analysis of NF-κB Pathway Activation

We examined the NF-κB signaling pathway activation using the Milliplex map NF-κB signaling magnetic beads kit 6-plex cell signaling assay (Millipore Sigma, 48-630MAG). Untreated or treated cells were lysed in Milliplex map lysis buffer containing protease inhibitors. Each lysate was diluted in assay buffer according to the manufacturer’s instructions and frozen at −80 °C until the protein measurements were performed. Samples were processed according to the assay protocol, and the median fluorescence intensity (MFI) was measured with a Luminex system.

### Cytokine Measurement

Cytokines were quantitatively determined using MILLIPLEX Human Cytokine/Chemokine/Growth Factor Panel A (Cat. No. HCYTA-60K). Samples were analyzed on a Luminex 200 System with MILLIPLEX Analyst 5.1 software. Data analysis was performed for all immunoassays using the Belysa Immunoassay Curve Fitting Software (Cat. no. 40-122). Figures were prepared in GraphPad Prism.

### Statistical Analysis

All values are expressed as the mean ± SEM of the mean and are representative of two to three independent experiments as indicated. P values were calculated using unpaired two-tailed Student’s tests, built in the GraphPad Prism Software for evaluation of statistical significance.^50^

## Supplementary Material



## Notes

VOC classifications and concentrations; VOC effects on the NF-κB pathway by cell type; qPCR validation of NFKB1 knockout; and authors and affiliations (PDF)
