# Supplementary material for: Encapsulation of Exosomal Volatile Organic Compounds for Modulating the NF-κB Pathway in Monocytes
Source: ACS Bio Med Chem Au. 2026 Mar 16;6(3):237–53. doi: 10.1021/acsbiomedchemau.5c00241 (PMC13281010; doi:10.1021/acsbiomedchemau.5c00241)
Supplement: Supplementary file 1 [file bg5c00241_si_001.pdf]

Encapsulation of Exosomal Volatile Organic Compounds for Modulating the NF-κB Pathway in Monocytes

Dina Hashoul[1,3] Rand Shibel[1,3], Haya Dahamshy-Silawi[2], Elias Mansour[1], Nisrine Lahoud Jeries[2], Walaa Saliba[1], Yoav Y. Broza[1], Assaf C. Bester,[2] and Hossam Haick[1*]

[1] *Department of Chemical Engineering and Russell Berrie Nanotechnology Institute, Technion - Israel Institute of Technology, Haifa 320003, Israel.*

[2] Faculty *of Biology, Technion - Israel Institute of Technology, Haifa 320003, Israel.*

[3] Authors contributed equally to this work.

[*] *Correspondence and requests should be addressed to H.H. (E-mail: hhossam@technion.ac.il)*

<u>Supplementary information</u>

Supporting Information S1: Table S1 VOCs classifications and concentrations

Compound	CAS No.	Classification	U937 Concentration-µM	KO Concentration-µM	KO+LPS Concentration-µM	U937+LPS Concentration-µM	U937 Concentration-ppb	KO Concentration-ppb	KO+LPS Concentration-ppb	U937+LPS Concentration-ppb	RSD%	R2	LOD-ppb
Styrene	100-42-5	Aromatic compounds	0.01	0.03	0.017	0.034	1.04	3.12	1.76	3.536	14.25	0.97	1#
Hexanal	66-25-1	Aldehydes	3.6	4.1	2.67	4.2	360.00	410	267	420	7.58	0.99	
2-ethyl-hexanol	104-76-7	Alcohols	1.54	2.56	0.98	3.74	200.20	330	127	480	21.17	0.98	
Heptanone	110-43-0	Ketones	0.187	0.32	0.192	0.361	18.70	32	19.2	36.1	10.98	0.98	
Butanal	123-72-8	Aldehydes	0.241	0	0.274	0	17.35	0	19.728	0	8.67	0.99	
Octanal	124-13-0	Aldehydes	2.13	4.03	4.1	4.35	272.64	515.84	524.8	556.8	4.36	0.99	
Cyclohexanone	108-94-1	Ketones	0.03	0.014	0.036	0.018	2.94	1.372	3.528	1.764	6.22	0.97	
Propanol	71-23-8	Alcohols	0.057	0.023	0.063	0.037	3.42	1.38	3.78	2.22	14.02	0.99	
2-butanone	78-93-3	Ketones	11.46	6.01	12.34	4.85	825.12	432.72	888.48	349.2	15.26	0.98	

Supporting Information S2: Table S2 VOCs classifications and concentrations

Compound	CAS No.	Classification	U937 Concentration-µM	KO Concentration-µM	KO+LPS Concentration-µM	U937+LPS Concentration-µM	A549 Concentration-µM	U937 Concentration-ppb	KO Concentration-ppb	KO+LPS Concentration-ppb	U937+LPS Concentration-ppb	A549 Concentration-ppb	RSD%	R2	LOD-ppb
Octanal	124-13-0	Aldehydes	0.24	0.44	1.65	2.03	0.61	30.72	0.05632	211.2	259.84	78.08	11.40	0.98	1#
2-butanone	78-93-3	Ketones	5.03	5.43	0	0	2.96	362.16	390.96	0	0	213.12	9.20	0.99	
3-hexanone	589-38-8	Ketones	0.016	0.015	0.0202	0.0205	0.0402	0.001152	1.5	1.6	2.02	4.02	5.30	0.98	
octanol	111-87-5	Alcohols	0.027	0.036	0.051	0.062	0.042	3.51	4.68	6.63	8.06	5.46	15.60	0.97	

Supporting Information S3: Table S3 VOC Effects on NF-κB Pathway by Cell Type

VOC	Cell Type	NF-κB (p-p65)	IKKα/β	IκBα (p-IκBα)	TNFR1	FADD (p-FADD)	C-MYC	Notes
2-Butanone	WT U937	↓↓↓	↑↑	↓↓↓ (+LPS)	↓↓↓ (+LPS)	Not shown	Not shown	Strong NF-κB downregulation in both WT and KO cells; upstream IKK activation with reduced IκBα phosphorylation.
	KO U937	↓↓↓	↓ (-LPS)	↓↓↓	↓↓↓			
1-Butanol	WT U937	Not shown	↑	↑↑ (-LPS)	↑ (-LPS)	↑↑ (+LPS)	↑↑↑ (+LPS)	Moderate activation in WT U937; prominent FADD & C-MYC upregulation, TNFR1 unchanged.
	KO U937		↑	↔	↔	↑ (-LPS)	↑↑	

3-Hexanone	WT U937	↔	↑↑ (+LPS)	↑↑↑	↔	Not shown	Not shown	Similar profile to 1-Butanol in WT U937; less potent overall.
	KO U937	↑ (-LPS)	↑↑↑ (-LPS)	↑ (-LPS)	↑ (+LPS)			
Octanol	WT U937	↑↑↑	↑↑	↑↑↑	Not shown	↑↑	↑	Moderate to strong activation in WT and KO cells; FADD and C-MYC moderately increased.
	KO U937	↑	↑	↑↑		↑↑↑	↑	
Octanal	WT U937	↓↓↓ (-LPS)	↓↓↓ (-LPS)	↑↑(+LPS) ↓↓↓ (-LPS)	↑↑(+LPS) ↓↓↓ (-LPS)	↑↑↑↑	↓↓↓ (-LPS)	Divergent response: strong activation in WT with LPS stimuli, suppression in KO cells.
	KO U937	↓↓↓	↓↓↓ (-LPS)	↓↓↓ (-LPS)	↓↓↓	↓ (-LPS)	↓	

Supporting Information S4: Figure S4 NFKB1 knock-out

Gene expression:

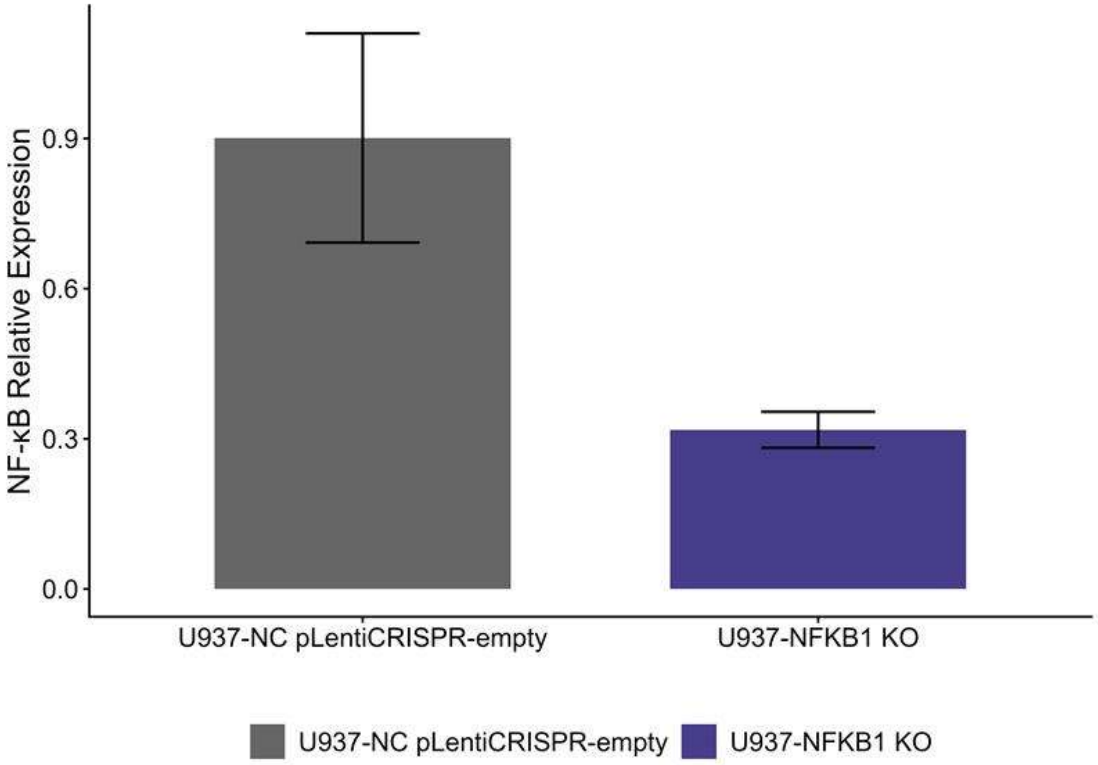

Figure S4. qPCR validation of *NFKB1* knockout: Relative mRNA expression of *NFKB1* was measured by quantitative PCR in control (Ctrl) and *NFKB1* knockout (NFκB KO) samples. Transcript levels were normalized to an internal housekeeping gene and expressed as relative quantity (RQ) using the ΔΔCt method, with control samples set to 1. Bars represent mean RQ values, with error bars indicating variability among replicates. *NFKB1* expression is markedly reduced in NFκB KO samples compared to controls, confirming efficient gene knockout.

Supporting Information S5: List of authors and affiliations

Dina Hashoul[1,3]	*1 Department of Chemical Engineering and Russell Berrie Nanotechnology Institute, Technion - Israel Institute of Technology, Haifa 320003, Israel.*	dinahashoul@gmail.com
Rand Shibel[1,3]	*1 Department of Chemical Engineering and Russell Berrie Nanotechnology Institute, Technion - Israel Institute of Technology, Haifa 320003, Israel.*	rand.shibel@mail.huji.ac.il
Haya Dahamshy Silawi[2]	2 Faculty *of Biology, Technion - Israel Institute of Technology, Haifa 320003, Israel.*	hayad@campus.technion.ac.il
Elias Mansour[1]	*1 Department of Chemical Engineering and Russell Berrie Nanotechnology Institute, Technion - Israel Institute of Technology, Haifa 320003, Israel.*	eliasm@campus.technion.ac.il
Nisrine Lahoud Jeries [2]	2 Faculty *of Biology, Technion - Israel Institute of Technology, Haifa 320003, Israel.*	nisrinelahoud@technion.ac.il
Walaa Saliba[1]	*1 Department of Chemical Engineering and Russell Berrie Nanotechnology Institute, Technion - Israel Institute of Technology, Haifa 320003, Israel.*	walaasaliba@gmail.com
Yoav Y. Broza[1]	*1 Department of Chemical Engineering and Russell Berrie Nanotechnology Institute, Technion - Israel Institute of Technology, Haifa 320003, Israel.*	yybroza@technion.ac.il
Assaf C. Bester[2]	2 Faculty *of Biology, Technion - Israel Institute of Technology, Haifa 320003, Israel.*	bestera@technion.ac.il
Hossam Haick[1]	*1 Department of Chemical Engineering and Russell Berrie Nanotechnology Institute, Technion - Israel Institute of Technology, Haifa 320003, Israel.*	hhossam@technion.ac.il
